# Awareness and knowledge of glaucoma and associated factors among adults: a cross sectional study in Gondar Town, Northwest Ethiopia

**DOI:** 10.1186/s12886-017-0542-z

**Published:** 2017-08-24

**Authors:** Destaye Shiferaw Alemu, Alemayehu Desalegn Gudeta, Kbrom Legesse Gebreselassie

**Affiliations:** Department of Optometry, College of Medicine and Health Sciences, University of Gondar, P.O. BOX: 196 Gondar, Ethiopia

**Keywords:** Awareness, Glaucoma, Knowledge, Gondar, Ethiopia

## Abstract

**Background:**

Raising public awareness and knowledge about glaucoma is a key for early case identification and prevention of blindness. However, awareness and knowledge about glaucoma is unknown at community level, making provision of interventions difficult. This study was intended to assess the awareness and knowledge of adults about glaucoma and the factors affecting it in Gondar town, Northwest Ethiopia.

**Methods:**

Community based cross - sectional study was conducted on 701 adults 35 and above years in Gondar from April 12–30, 2016. Multistage sampling technique was used to select study participants. Interviewer administered pretested structured questionnaire was used to collect data after verbal informed consent. Data were entered into EpiData version 3.1 and analyzed by Statistical Package for Social Sciences version 20. Bivariate and multivariate logistic regression models and Odds ratio with 95% interval were used to identify factors. P–value <0.05 was considered statistically significant.

**Results:**

Seven hundred one adults age 35 and above years were participated with a response rate of 99.3%. The male to female ratio was 1:1.6 with median age of 48 years with interqurtile range of 20. The proportion of awareness was 35.1% (95% CI: 31.5%, 38.6%). Good knowledge was demonstrated in 49.6% (95%CI: 43.3%, 55%) of glaucoma aware participants. Education (primary [AOR: 3.21; 1.73, 5.95], secondary [AOR: 4.34; 2.30, 8.22]; college and above [AOR: 9.82; 4.27, 22.60]) and having eye examination [AOR: 2.78; 1.86, 4.15] were positively associated with awareness of glaucoma whereas older age (65 –74 years [AOR: 0.31(0.21, 0.76]) was inversely related. Level of Education (primary[AOR:2.83;1.04,7.71],secondary[AOR:3.45;1.33,9.41],college and above [AOR: 4.86;1.82,12,99] and having eye examination [AOR: 2.61;1.53,4.45] were significantly associated with knowledge.

**Conclusion:**

The study has indicated higher level of awareness and knowledge about glaucoma in urban communities than previous studies. It has also identified educational status, eye examination at least once in life are related with better awareness and knowledge. The present awareness and knowledge should be enhanced through public oriented glaucoma education via mass media and incorporating eye check up as a routine in older people.

**Electronic supplementary material:**

The online version of this article (doi:10.1186/s12886-017-0542-z) contains supplementary material, which is available to authorized users.

## Background

Glaucoma is a group of eye diseases with characteristic features of optic disc and specific pattern of visual field defects [1]. The process of optic nerve damage is usually progressive and asymptomatic [2]. Intraocular pressure is the only modifiable risk factor [3] and other several risk factors have been identified [4–8].

Primary open-angle glaucoma (POAG) is the most common type of glaucoma in whites and Afro-Caribbean accounting 2% of visual impairment and 8% of blindness [9].

Glaucoma is a non - communicable, chronic eye disease which needs the principles of long-term care [10].

The asymptomatic nature along with the irreversible blindness it causes makes glaucoma a public health challenge [11] and the second cause of avoidable blindness globally [12]**.**

Approximately, 15% of global blindness is due to glaucoma and around 600,000 people go blind annually [13]. In 2010, 60.5 million people were victims of glaucoma globally [14]. This is expected to rise to 76 million in 2020 and 111 million in 2040 [15].It was also reported that 57.5 million people were affected in 2015 by POAG alone [16].

Blindness due to glaucoma is highest in Africa [14] accounting 15% [17] of the global blindness (4.20%) [18]. The situation is worse in Sub Saharan Africa [17] where poor awareness and knowledge further compounded the condition. In Ethiopia, glaucoma is the fifth cause of blindness causing an irreversible sight loss for an estimated of 62,000 Ethiopians [19].

Glaucoma blindness imposes significant economic burden not only for individuals affected [20] but also it increases healthcare cost [21], impairs quality of life, increases rehabilitation cost for the blind which all affects the economic growth of a nation [22]. It also results a huge burden for the healthcare system and government’s spending toward health care [23].

Early detection and timely treatment are important to avert the consequences from such silent thief of sight [24]. As most cases occur among the productive age group, preventing early blindness from glaucoma is basically not only a matter of saving individuals sight loss but also saving nations economy [25].

Public awareness and knowledge of glaucoma plays a significant role in raising public health seeking behavior for regular eye check and increases the chance of identifying undetected cases [26]. It is also clinically beneficial and cost effective to delay visual field deterioration [27, 28] and improve treatment compliance [12, 29, 30]. Contrary to this, failure to aware leads to late detection and poses management problem in preventing blindness from glaucoma [13]. Despite this fact, poor public awareness and knowledge about glaucoma is the major gap. In the developed world, less than 50% of people with glaucoma are aware of it [31], almost 70% of cases are not detected [32] and 39% of them present with advanced stage of the disease in at least one eye [33]. This is worse in developing countries where few people are aware and knowledgeable about glaucoma [34, 35].

Raising public awareness and knowledge of glaucoma is a key means of addressing the overwhelming consequences of the disease [2, 36, 37].Cognizant to this, international directives [38] including the World Health Organization’s Vision 2020 campaign [12] took glaucoma one of its main priority area.

It would appear that the global Vision 2020 initiative has placed glaucoma as a seventh eye disease priority [39]. The World Glaucoma Association (WGA) and the World Glaucoma Patient Association (WGPA) extended ‘World Glaucoma Day’ to ‘World Glaucoma Week (WGW)’ to help people understand the distressing effects of the disease [14]. In Africa, the WGA 1st Africa glaucoma summit in Ghana in 2010 [40] and the Kampala, Uganda, resolution in 2012 [41] were some to mention some.

In Ethiopia, efforts have been done to raise public awareness of glaucoma. In 2007, a group of volunteers consisting of physicians and glaucoma patients set up the Glaucoma Group aimed at increasing public awareness of glaucoma supported by the ophthalmological Society of Ethiopia (OSE) and the ministry of health every year, during World Glaucoma Week [24]. However, evidence on public awareness and knowledge about glaucoma is limited in Ethiopia except a few institution-based studies in some parts of the country. These pocket studies may not reflect the real picture of awareness and knowledge in the public about glaucoma as they were few and mainly institution based. Hence, this study was aimed to investigate the awareness and knowledge of adults towards glaucoma and associated factors in Gondar town, Ethiopia.

There is a widely accepted notion that awareness leads to knowledge and knowledge to behavior [42]. However, published evidences indicates that awareness and knowledge about glaucoma is limited across the globe [40–61].

Various factors influence awareness and knowledge about glaucoma. In the available literatures level of education [13, 40, 41, 44, 46, 49, 51, 61–63], family history of glaucoma [46, 63], age [40], economic status ([13, 40, 48, 51]), sex [44, 48], type of occupation [51, 64], having chronic disease like diabetes and hypertension [20] and having eye examination [64] were associated with awareness and knowledge of glaucoma.

Public awareness and knowledge plays a paramount role for early detection and timely treatment of glaucoma. This delays early blindness from glaucoma. It is also equally important to design most effective awareness raising strategies based on identified factors affecting the awareness and knowledge about glaucoma. There is, however, as far as our web site database search was concerned, paucity on awareness and knowledge about glaucoma and the associated factors in Ethiopia in general and the study area in particular. The present study intended to fill this gap while serving as a baseline for further studies to generate inputs for eye health care providers and policy makers to design evidence based interventions to lessen the alarmingly increasing blindness from glaucoma.

## Methods

### Study design and study area

A community based cross- sectional was study carried out in Gondar town, Northwest Ethiopia. Gondar has an estimated population of 210,000 (47.4% males, 52.6% females) [65] and is one of the largest towns of the country. The town has 21 kebeles (smallest administrative units in Ethiopia) with 53,725 households [65]. There is a referral University hospital with a tertiary eye care center. The eye care center provides general and various specialty ophthalmic services, including glaucoma. There are also few private ophthalmic clinics in the town, which provide general eye care services. The study period was from April 12–30, 2016***.***

### Source and study population

The source population for the study was all adults (age 35 and above years) in Gondar town (This segment of the population was targeted for the study to reflect the awareness and knowledge of glaucoma among the population most at risk).

### Inclusion and exclusion criteria

All permanent residents (lived at least 6 months) in the study area whose age was 35 and above years were eligible to participate in the study while permanent residents age 35 and above years who were eye care professionals and individuals who were seriously ill to answer the study questions were excluded.

### Variables of the study

The outcome variables were awareness and knowledge of glaucoma while the independent variables include *socio – demographic factors*: age, sex, religion, marital status; *socio-economic factors*: educational level, type of occupation, income; *health related factors*: history of diabetes, history of hypertension, family history of glaucoma, history of eye examination.

### Operational definitions

#### Aware

A participant was classified as aware of glaucoma if a positive response (‘Yes’) was obtained to the question *‘have you ever heard of glaucoma*?’ and gave at least one answer such answers as ‘glaucoma is high eye pressure’, ‘glaucoma is high eye pressure causing blindness’, ‘glaucoma causes damage to the eye nerve’, blinding eye disease causing eye nerve damage, eye disease cause visual field loss [60]. In this study, hearing glaucoma alone was not considered as awareness because merely being aware of the term did not ensure awareness about the disease. In a previous study, on a similar topic in Ethiopia [60] and the pretest procedure of this study indicated that participants who said “Yes” for the question *‘have you ever heard of glaucoma*?’ were meant to them ‘trachoma’. Probably trachoma is common in the study area and the two conditions, “Trach**oma**” and “Glauc**oma**” have similar suffix (‘-**oma**’) as well as there is no common Amharic (local language term) equivalent for glaucoma.

#### Knowledge

Respondents who scored the mean (≥8.42) and above of the knowledge questions were considered to have good knowledge while those who score below the mean were considered as having poor knowledge.

#### Scoring

Fifteen questions adapted from previous studies [44, 60], were used to assess respondents’ knowledge about glaucoma. One point was allocated for each correct response; otherwise, zero was given.

### Sample size determination

Sample size was determined separately for awareness, knowledge and factors to address the objectives. The single population proportion formula was considered to address the first two objectives with the assumption of 95% confidence level (Z_ɑ/2_ = 1.96), 5% maximum allowable error (w), 10% non-response and a design effect 2 for multistage sampling. Accordingly, the sample size required for awareness (considering proportion of awareness *p* = 0.284 ( [61]) was 689. The calculated sample size for knowledge based on the proportions of,*p* = 0.703 for good knowledge [61] was 706. The power approach, using StatCalc (Epi info 7), was used to calculate sample size to address the objectives for factors based on variables which were statistically significant in previous studies (educational level, age, sex) [61]. Educational status was the most repeatedly statistically significant variable and sample size was determined considering a power of 80%, non- exposed to exposed ratio 1:1, confidence level of 95%, design effect of 2 for multistage sampling and 10% for non-response. The sample size based on this factor was 185 and for the other two was 156 (for age) and 110 (for sex). The final sample size required for the study was 706 adults (Additional file 1).

### Sampling technique

Multistage sampling technique was used for the sampling process. Initially, 20% of the total “kebeles” were selected using simple random sampling technique after obtaining their list from the local administration office. The required sample from each kebele was allocated according to the proportion to size of each kebele population. The sampling fraction (k = 22) was determined by taking the ratio of households in the respective selected kebele to the sample size selected in each kebele. (i.e. k = Ni/ni, where Ni = total population in each selected kebele and ni = sample size taken from each selected kebele). Then, households were selected using systematic random sampling technique. Finally, one eligible adult was selected from each household using simple random sampling. The next immediate household was taken when no person selected fulfilled the eligibility criteria of the study (Additional file 2).

### Data collection tool

The questionnaire was adapted from previous studies [44, 60]. It was initially prepared in English by the principal investigator, translated into Amharic (local language) by language expertise, and re-translated to English to check consistency in meaning of words and concepts. Most questions were closed ended. The questionnaire consisted of background information and questions to measure awareness and knowledge of glaucoma (Additional file 3). It was pretested and modified prior to actual data collection.

### Data collection procedures

Before starting data collection, from which direction to start was determined by tossing a coin being at a prominent landmark (road, big buildings). The first household was the first number between one and the sampling fraction (k = 22).

Then data collectors (five BSc optometrists) contacted the house head and the study participant and obtained permission to proceed interviewing using Amharic version of the questionnaire. Study participants who responded for the awareness questions were further asked the knowledge questions. Weekends were preferred to other days for data collection to maximize the chance of getting study participants (Additional file 4).

### Data quality assurance

To assure quality, one-day training was given to data collectors and supervisors by the principal investigator on how to use the questionnaire and the data collection procedure as well as the sampling technique. The questionnaire was pretested prior to the actual data collection on 5% of the sample [35] who fulfilled the sampling inclusion criteria in an area with similar characteristic outside the study area (Teda town). Modifications were made accordingly. The supervisor and principal investigator supervised data collection process. The completeness, accuracy and clarity of data were checked on daily bases. EpiData version 3.1 was used for data entry after coding.

### Data management and analysis

Data were entered into EpiData version 3.1 and exported to Statistical Package for Social Science (SPSS) version 20 for analysis. Data were cleaned using frequencies and cross tabulations. Data were described using summary measures (frequencies, proportions means). Proportions were estimated along with 95% CI. Both bivariate and multivariate logistic regression analyses were carried out. Variables with *p* value <0.2 in the bivariate analysis were fitted into the multivariate logistic regression model for prediction of determinants. Enter method was used for variable selection. The Hosmer-Lemeshow goodness-of- fit statistic (0.76) was used to assess whether the necessary assumptions for the application of multiple logistic regression were fulfilled. Crude and adjusted odds ratio with 95% confidence interval were computed. The Adjusted Odds ratio with 95% confidence interval was used to measure the strength of association and the actual predictors of the outcome variables. *P*-values less than 0.05 were considered statistically significant.

## Results

### Socio – Demographic characteristics of study population

A total of 706 adults age 35 and above years were initially planned to participate in the study, however; five participants refused to participate, and were excluded from the study giving a response rate of 99.3%. The sex proportion of the participants was almost equal with male to female ratio of 1:1.6. The median (± IQR) age was 48 (±20) years.

Majority of the respondents were Orthodox Christian in religion (77.6%) and Amhara in ethnicity (84.9%). More than two-third (68.3%) were married and less than one – third (30.1%) were housewives. About two out of five participants (42%) were in the age group 35 – 44 years and almost one – third had no formal education (32.7%) (Table 1).

**Table 1:** Socio–demographic characteristics of study population, Gondar town, Northwest Ethiopia, April 2016 (*n* = 701)

Characteristics	Frequency	Percent
Sex		
Female	376	53.6
Male	325	46.4
Age (years)		
35 - 44	296	42.1
45 - 54	163	23.3
55 - 64	112	16.0
65 - 74	74	10.6
≥ 75	56	8.0
Religion		
Orthodox	544	77.6
Muslim	142	20.2
Others*	15	2.2
Ethnicity		
Amhara	595	84.9
Tigrie	51	7.3
Kmant	49	7.0
Others**	6	0.8
Level of education		
No formal education	229	32.7
Primary education	187	26.6
Secondary education	166	23.7
College and above	119	17.0
Marital status		
Single	53	7.6
Married	479	68.3
Divorced	59	8.4
Widowed	110	15.7
Type of occupation		
Merchant	216	30.8
Housewife	211	30.1
Government employed	115	16.4
Self-employed	55	7.8
No job	37	5.3
Farmer	21	3.0
Daily laborer	20	2.9
Others***	26	3.7
Monthly income in ETB (*n* = 617)		
≤ 750	174	28.2
751-1300	116	18.8
1301-2000	130	21.1
> 2000	197	31.9

Others*protestant, Jewish; Others**Oromo, Guragie; Others***retired, driver, waiver, religious leaders, ETB: Ethiopian birr

### Medical and glaucoma related characteristics in the study population

Ninety-three (13.3%) study participants had self-reported history of established hypertension, yet 35(37.6%) of them never examined their eyes. Among 58(8.3%) respondents with diabetes mellitus, 22 (37.9%) of them had never eye checkup in their life. Among respondents with family history of glaucoma [21], 38.1% [8] had never eye examination. Two hundred sixty – one (37.2%) of the respondents had history of eye examination at least once in their lifetime. Among these, about three fourth had at least one examination in the last 12 months and one fourth of them had before 12 months (Table 2).

**Table 2:** Medical and glaucoma related characteristics of study population, Gondar town, Northwest Ethiopia, April 2016

Characteristics	Frequency	Percent
History of hypertension (*n* = 701)		
Yes	93	13.3
No	551	78.6
Do not know (not screened)	57	8.1
History of diabetes (*n* = 701)		
Yes	58	8.3
No	582	83.0
Do not know	61	8.7
History of eye examination (*n* = 701)		
Yes	261	37.2
No	440	62.8
Eye examination (*n* = 261)		
Within 12 months	194	74.3
Before 12 months	67	25.7
History of glaucoma (*n* = 261)		
Yes	18	6.9
No	223	85.4
Do not know	20	7.7
Family history of glaucoma (*n* = 246)		
Yes	21	8.5
No	177	72.0
Do not know	48	19.5
Family member with glaucoma (*n* = 21)		
Mother	9	42.9
Brother	5	23.8
Father	4	19.0
Sister	2	9.5
Father and brother	1	4.8

Table 2: Medical and glaucoma related characteristics of study population, Gondar town, Northwest Ethiopia, April 2016.

### Awareness of adults towards glaucoma

**Fig. 1:**
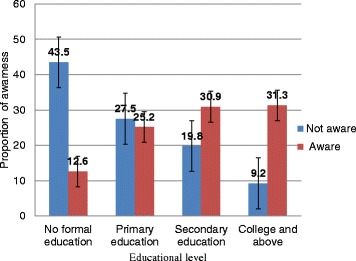
Proportion of awareness of glaucoma across educational level of respondents with 95% CI error bar among adults age 35 and above years, Gondar town, Northwest Ethiopia, 2016(*n* = 701)

Three hundred forty (48.5%) respondents had heard of glaucoma. Nevertheless, it was only 246 (35.1% [95% CI: 31.5%, 38.6%]) of them were aware of glaucoma. The mean age for respondents who were aware of glaucoma was 51.73 (SD ± 14.34) and for those who were not aware was 47.95 (±11.74) (*p* < 0.001). Higher proportions (43.52%) of adults without formal education were not aware of glaucoma (Fig. 1).

### Source of information about glaucoma

**Fig. 2:**
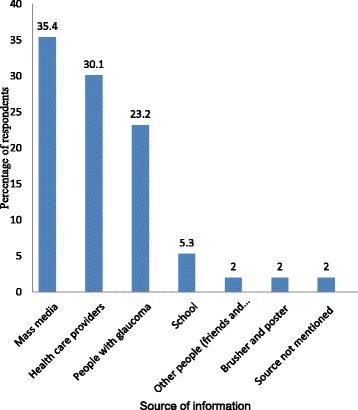
Sources of information about glaucoma among adults, Gondar town, Northwest Ethiopia, April 2016 (*n* = 246)

The respondents had multiple sources of information about glaucoma. Mass media (television and radio) followed by health care providers and people with glaucoma were the main sources of information (Fig. 2).

### Knowledge of glaucoma among respondents who were aware of glaucoma

Among 246 study participants who were aware of glaucoma, 122 (49.6% [95% CI: 43.33% - 56.9%]) had good knowledge about glaucoma while 124(50.4%) had poor knowledge. The mean knowledge score of glaucoma was 8.42 (±2.7 SD) with a maximum possible score of 15. Seventy-three (29.7%) males, 89(36.2%) married, 22(8.9%) hypertensives, 15(6.1%) diabetics, 77(31.3%) with eye examination and 6 (4.8%) respondents with glaucoma and 9(3.7%) with family history of glaucoma had good knowledge (Table 3).

**Table 3:** Knowledge of glaucoma across socio- demographic characteristics of respondents, Gondar town, Northwest Ethiopia, April 2016 (*n* = 246)

Characteristics	Knowledge status	
	Good N (%)	Poor N (%)
Sex		
Male	73(29.7)	64(26.0)
Female	49(19.9)	60(24.4)
Age (years)		
35 – 44	50(20.3)	62(25.2)
45 – 54	38(15.4)	31(12.6)
55 – 64	24(9.8)	18(7.3)
65 – 74	6(2.4)	4(1.6)
≥ 75	4(1.6)	9(3.8)
Level of education		
No formal education	7(2.8.)	24(9.8)
Primary education	28(11.3)	34(13.8)
Secondary education	41(16.7)	35(14.3)
College and above	46(18.7)	31(12.6)
Religion		
Orthodox	94(38.2)	94(38.2)
Muslim	25(10.2)	27(11.0)
Others*	3(1.2)	3(1.2)
Ethnicity		
Amhara	99(40.2)	105(42.7)
Tigrie	9(3.7)	10(4.1)
Kmant	13(5.3)	6(2.4)
Others**	1(0.4)	3(1.2)
Marital status		
Single	12(4.9)	6(2.4)
Married	89(36.2)	97(39.4)
Divorced	12(4.9)	10(4.0)
Widowed	9(3.7)	11(4.5)
History of hypertension		
Yes	22(8.9)	22(8.9)
No	98(39.8)	93(37.8)
Do not know (unscreened)	2(0.8)	9(3.8)
History of diabetes mellitus		
Yes	15(6.1)	12(4.9)
No	104(42.3)	106(43.1)
Do not know (unscreened)	3(1.2)	6(2.4)
History of eye examination		
Yes	77(31.3)	49(19.9)
No	45(18.3)	75(30.5)
History of glaucoma***		
Yes	6(4.8)	7(5.6)
No	69(54.8)	38(30.2)
Do not know	2(1.6)	4(3.2)
Family history of glaucoma		
Yes	9(3.7)	5(2.0)
No	106 (43.2)	78(31.6)
Do not know	34(13.8)	14(5.7)

Others* = Protestant & Jewish, Others** = Oromo &Guragie

### Factors associated with awareness of glaucoma

In this study age, sex, marital status, educational level, type of occupation, history of hypertension, history of diabetes mellitus and having had eye examination were associated with awareness of glaucoma at 0.2 level of significant in the binary logistic regression analysis.

However, after adjusting for potential confounders in the multivariate analysis, educational level (primary education [AOR: 3.21; 1.73, 5.95], secondary education [AOR: 4.34; 2.30, 8.22], college and above [AOR: 9.82; 4.27, 22, 60]) and having had eye examination at least once in life (AOR: 2.78; 1.86, 4.15) were directly associated with awareness of glaucoma. Nevertheless, age (group 65 – 74 [AOR: 0.31; 0.21, 0.76]) was inversely associated.

Accordingly, adults with primary education were 3.2 times more likely to be aware of glaucoma compared with adults with no formal education. Similarly, adults with secondary education were 4.34 times more aware of glaucoma compared with adults with no formal education. Furthermore, adults with college and above education were nearly ten times more likely to be aware of glaucoma than adults without formal education. Adults with at least one eye examination in their life were nearly three times more likely to be aware of glaucoma than adults who had never examined their eyes.

People whose ages between 65 and 74 years were 69% less aware of glaucoma compared with their counterparts in the age range 35–44 years. Awareness of glaucoma was independent of, sex, marital status, type of occupation, history of hypertension and history of diabetes mellitus (Table 4).

**Table 4:** Bivariate and multivariate logistic regression of factors associated with awareness of glaucoma among adults age 35 and above years, Gondar town, Northwest Ethiopia, April 2016 (*n* = 701)

Variables	Awareness			
	Aware	Not aware	COR (95%CI)	AOR (95%CI)
Age category (years)				
35 - 44	112	184	1.00	1.00
45 - 54	89	74	1.21(0.82,1.78)	1.27(0.78,2.10)
55 – 64	54	58	0.99 (0.63,1.54)	1.29(0.72,2.32)
65 – 74	27	47	0.26(0.13,0.52)***	0.31(0.21,0.76)*
≥ 75	17	39	0.50(0.26,0.96)*	0.81(0.32,2.05)
Sex				
Male	137	188	1.00	1.00
Female	109	267	0.56(0.41-0.77)***	0.74(0.46,1.20)
Level of education				
No formal education	31	198	1.00	1.00
Primary education	62	125	3.17(1.95,5.15)***	3.21(1.73,5.95)*
Secondary education	76	60	5.39(3.32,8.77)***	4.34(2.30,8.22)**
College and above	77	42	11.71(6.87,19.9)***	9.82(4.27,22.60)***
Marital status				
Single	18	35	1.23(0.68-2.24)**	0.93(0.45,1.92)
Married	186	293	1.16(0.53-2.51)*	1.30(0.49,3.43)
Divorced	22	37	0.43(0.21-0.91)***	1.04(0.39,2.81)
Widowed	20	90	1.00	1.00
Type of occupation				
Merchant	90	126	1.00	1.00
Housewife	45	166	0.38(0.25-0. 58)***	0.92(0.51,1.67)
Government employed	66	49	1.89(1.19-2.98)**	0.92(0.48,1.74)
Others	33	7143	0.67(0.32-1.41)	2.33(0.78,6.9)
Self-employed	12		0.39(0.20-0.78)**	0.50(0.23,1.10)
Hypertension status				
Yes	44	49	1.69(1.09-2.64)*	1.43(0.21,9.73
Do not know	11	46	0.45(0.23-0.89)**	0.82(0.12,5.47)
No	191	360	1.00	1.00
Diabetes mellitus status				
Yes	27	31	1.54(0.90-2.66)	2.34(0.36,15.21)
Do not know (unscreened)	9	52	0.31(0.15-0.64)**	1.92(0.31,11.96)
No	210	272	1.00	1.00
Eye examination				
Yes	126	135	2.49(1.81-3.3)***	2.78 (1.86,4.15)***
No	120	320	1.00	1.00

*COR* adjusted odds ratio, *AOR* adjusted odds ratio, *CI* confidence interval

*p-value <0.05, **p-value **<**0.01, ***p-value <0.001, 1 = reference, others: retired, driver, religious leader, farmer, no job daily laborer

### Factors associated with knowledge of glaucoma

Sex, educational status and history of eye examination were significantly associated with good knowledge in the bivariate logistic regression analysis. Educational status and having eye examination at least once in life are independent predictors of good knowledge after controlling potential confounders.

Accordingly, adults with primary education were nearly three times (AOR: 2.83; 1.04, 7.71) more likely to have good knowledge than adults without formal education. Similarly, the likelihood of having good knowledge among adults with secondary education was more than three times (AOR: 3.53; 1.33, 9.41) higher than adults without formal education. Furthermore, the likelihood of having good knowledge among adults with college and above education was almost five times (AOR: 4.86; 1.82, 12.99) higher than those adults without formal education. The likelihood of good knowledge about glaucoma among adults who had at least one eye examination in their life was 2.6 times higher (AOR: 2.61; 1.53, 4.45) compared to adults who had never an eye examination in their life (Table 5).

**Table 5:** Bivariate and multivariate logistic regression of factor affecting good knowledge about glaucoma, Gondar town, 2016 (*n* = 246)

Variable	Knowledge		COR (95%CI)	AOR (95%CI)
	Good	Poor		
Age category (years)				
35 - 44	50	62	1.00	-
45 - 54	38	31	1.52(0.83,2.78)	
55 – 64	24	18	1.65(0.81,3.38)	
65 – 74	6	4	1.86(0.50,7.0)	
≥ 75	4	9	0.55(0.16,1.90)	
Sex				
Male	73	64	.00	1.00
Female	49	60	0.72(0.43,1.19)^**c**^	0.76(0.44,1.30)
Educational level				
No formal education	7	24	1.00	1.00
Primary education	28	34	2.82(1.06,7.52)*	2.83(1.04,7.71)*
Secondary education	41	35	4.02(1.55,10.44)**	3.53(1.33,9.41)*
College and above	46	31	5.09(2.00,13.25)**	4.86(1.82,12.99)**
Marital status				
Single	12	6	1.00	-
Married	89	97	0.46(1.65,2.17)**	
Divorced	12	10	0.60(0.17,2.17)	
Widowed	9	11	0.41(0.11,1.53)	
Diabetes mellitus status				
Yes	15	106	0.79(0.35,1.76)	-
Do not know	104	104	0.40(0.08,1.94)	
No	3	6	1.00	
Hypertension status				
Yes	22	22	1.05(0.55,2.03)	
Do not know(not screened)	98	93	0.22(0.43,1.15)	
No	2	9	1.00	
Eye examination				
Yes	77	49	2.62(1.57,4.38)***	2.61(1.53,4.45)***
No	45	75	1.00	1.00

*COR* crude odds ratio, *AOR* adjusted odds ratio, *CI* confidence interval

^c^ = *p* < 0.20, *p < 0.05, **p < 0.01, ***p < 0.001, 1 = reference

## Discussion

This population based cross sectional study determined the awareness and knowledge level of adults about glaucoma and the associated factors in Gondar town, Northwest Ethiopia.

In the present study, the proportion of awareness was 35.1% (95% CI: 31.5%, 38.6%) while good knowledge among respondents who were aware of glaucoma was 49.6% (95%CI: 43.3, 55). Educational status (having at least formal education), eye examination at least once in life were directly related to better awareness about glaucoma whereas older age was inversely related. Similarly, higher level of education and having eye examination at least once in life were determinants of good knowledge about glaucoma.

The proportion of awareness about glaucoma (35.1%) in the present study is higher than a finding from previous study in Ethiopia [61]. This is expected from such an urban-based population study where people have access to health related information at least due to geographic proximity to information [66].

The current level of glaucoma awareness (35.1%) is better as compared to reports from Agaro town in Jimma (2.4%), Southwest Ethiopia, in 2009 [60]. This may reflect the variation in demographic characteristics of the study population between the two studies. In the Agaro study, about 43% participants were not able to read and write while in the current study only 32.7% participants had no formal education. Additionally, participants in the former study were primarily rural residents coming for outreach service*.* This could be source of the discrepancy as people living in remote rural areas might have less chance of getting health related information [50]. Age difference among study participant might also be one possible explanation. In the Agaro study the participants were relatively aged (average 54.5) than participants in this study (average 48 years). Previous studies demonstrated older people are less aware of glaucoma [40, 44, 46, 61, 63].

This finding is also higher than reports from urban (4.8%) [20] and rural settings 13.5% [12],0.32% [53], 8.3%5 [54] of India which might be attributable to differences in study setting as well as socio-demographic differentials.

However, the finding of the present study is lower than the finding from urban population of Puducherry, India (45%) [44], Pakistan (48.7%) [50], Iran (46.6%) [52] and North-central Nigeria (47%) which might be due to the difference in the way awareness was measured. The present study has used a little bit ‘stricter’ definitions to measure awareness and knowledge of glaucoma. In those previous studies, a study participant were considered aware of glaucoma if the respondent was able to provide a ‘Yes’ response only for the question “Have you heard of glaucoma”. However, in the present study a participant was considered aware of glaucoma if a ‘Yes’ response was obtained to the question ‘Have you heard of glaucoma?’ and additional description such as ‘glaucoma is high eye pressure’, ‘glaucoma is high eye pressure causing blindness’, ‘glaucoma is high eye pressure causing damage to the eye nerve’ or similar explanation was obtained. Socio-economic variations could explain these differences. Other studies from Ghana (74%) [58], Nigeria (74.3%) [56] and Southwest Nigeria 68.6% [57] also showed higher findings than this study which could partially be explained by variation in participants characteristics. In the former study, participants were glaucoma patients who were aware due to exposure to glaucoma related information [67].

On the other hand, the present study finding is in line with a study from central Ethiopia, Addis Ababa, Menelik II hospital in 2010 (28.4%) [61]. This might be due to the similarity in socio-demographic characterstics of study pasrticipants, study design, setting. The former and the present study were urban based cross sectional studies and the study participants had similar socio-demographic characteristics with similar male to female participants ratio (1:1.1vs 1:1.6), age (40.5 vs. 48 years) and educational level.

From the three studies in Ethiopia, despite the different study population and settings, it appears that there is a paradigm shift of awarness about glaucoma. The due attention given to galucoma in the recent few years [24] may explain this. This could also be the effect of the expansion in eye care service through the increasing number of eye care professionals (optometrists and ophthalmologists) in Ethiopia [24].

The current study has also revealed better knowledge (49.6%) about glaucoma compared to similar studies in urban community of India 0.5% [12] and 34.04% [44], Ethiopia (12.1%) [61],South India (3.1%) [20] and Ghana (27%) [58]. This might be due to the difference methods used to measure knowledge across these studies. In the former studies the proportion of knowledge estimated from total study participants (both aware and not aware as a denominator) whereas in this study knowledge was estimated among those who were aware of glaucoma.

While the current finding is in line with an institution based study in Benin city, Nigeria (31%) [56], it was lower than a finding from Southwest Nigeria (88.3%) [57]. This might reflect the method used to estimate knowledge was different across these studies and the characteristics of the study participants was quite different from the present study. In the Benin City, Nigerian study measure knowledge was consistent in content (included risk factors, symptoms, treatment, and prevention of glaucoma) with the current study. Additionally, majority (77.8%) of the study participants had at least primary education, which is comparable with this study (67.8%).

In this study, people with at least primary education were better aware of glaucoma. Similar finding was also reported from Ethiopia [61] where better education was positively related with awareness of glaucoma. This could be due to the fact that the chance of exposure to different health related information education communication means increases as educational status increase [13, 41, 43, 44, 49, 51, 61–63].

Older individuals (age group 65 –74) were 69% less likely to be aware of glaucoma compared with those individuals in the age group 35 – 44. Similar finding [40] was also reported that where older people were less aware of glaucoma. However, in this study lower awareness was observed among adults between 65 and 74 years of age. The explanation for this may require a detailed study in this segment (age 65–74) of population. However, the current study found a smaller proportion of awareness of glaucoma (1.4%) among people age 65 – 74 years. Additionally, the proportion of risk factors for glaucoma including hypertension (1.12%) and diabetes mellitus (2.13%) were common and history of eye examination was reported in only 5.7% out of the total 701-study population. These findings may reflect that this segment of population is preoccupied with other general health issues.

It was also indicated that adults who had eye examination at least once in their life were more likely to be aware of glaucoma. Similar result was reported from Osun State, Nigeria [64].

According to this study, primary and above educational level was positively associated with better knowledge about glaucoma. This might be due to the higher number of literates, which may seek health related information that probably leads to awareness. In this study, 31.5% of the study participants were aware of glaucoma and above 62% were literates. In Indian study, knowledge increases exponentially among people with above college education [40]. Similar finding was also reported from other studies [13, 41, 44, 51, 63].

Eye examination at least once in life was related with good knowledge about glaucoma in this study. This is in line with a study from Osun State, Nigeria [64]. This might be due to the health education given for patients coming for eye examination help them to acquire some basic knowledge about the disease.

In previous studies, sex [44, 48] type of occupation [51, 64] history diabetes, hypertension [20], positive family history of glaucoma [46, 63] were significant determinants of awareness and knowledge of glaucoma. However, the data in the current study did not support this. This could be due to the small number of cases in this study.

### Strength of the study

Being community based, this study revealed the actual picture of public awareness and knowledge of glaucoma Using face-to-face interview allowed for clarification of misunderstood questions and reduced the frequency of missing items in the questionnaire.

### Limitations of the study

The study had some limitations that should be taken into account. It studied only residents who lived in households and homeless and people living in institutions (religious peoples, military, teachers and physicians) were not included which could either inflate or underestimate the reported finding in this study. Interviewer bias could not be eliminated as an individual’s expression, and style of explanation may affect the response of the participant. Data were also based on self-reporting which is subject to recall bias and income was not included in the analysis. The questionnaire was designed considering only primary open angle glaucoma. As a result, it was not possible to measure awareness and knowledge of people on other types of glaucomas. This study did not assess the attitude of participants towards glaucoma, which might have an impact on acquiring knowledge that might affect the knowledge assessment.

## Conclusion

The study has indicated the present level of awareness and knowledge among urban resident adults is high. It has also identified having at least primary education, eye examination at least once in life to be associated with better awareness and knowledge. It also identified mass media to be the main source of information about glaucoma.

## Additional files

Additional file 1: Sample size determination using the single population formula for awareness and knowledge (DOCX 13 kb)

Additional file 2: Sampling technique (DOCX 14 kb)

Additional file 3: English version of questionnaire (DOCX 26 kb)

Additional file 4: Data collection procedures (DOCX 13 kb)

## Abbreviations

CSA: Central Statistical Agency
OSE: Ophthalmological Society of Ethiopia
POAG: Primary open angle glaucoma
SPSS: Statistical Package for Social Sciences
