# Supplementary material for: Awareness and knowledge of glaucoma and associated factors among adults: a cross sectional study in Gondar Town, Northwest Ethiopia
Source: BMC Ophthalmol. 2017 Aug 24;17:154. doi: 10.1186/s12886-017-0542-z (PMC5571668; doi:10.1186/s12886-017-0542-z)
Supplement: Supplementary file 1 — Sample size determination using the single population formula for awareness and knowledge (DOCX 13 kb) [file 12886_2017_542_MOESM1_ESM.docx]

**Additional file 1: Sample size determination using the single population formula for awareness and knowledge**

The single population formula was used to estimate the minimum sample size, for both to address level of awareness and knowledge objectives

**n = Z_ɑ/2_p. q**

**w^2^** Where n = sample size, Z = value of z statistics at 95% confidence level = 1.96, w = maximum tolerable error, q = 1 – p.

Table showing sample size determination by using the single population proportion formula for knowledge

**Minimum sample size require to address awareness**

p= 0.284(proportion of awareness)

q = 1 – p = 0.716

W (maximum allowable error = 0.05)

n (sample size) = **689**

**Sample size required to determine good knowledge**

p = 0.703(proportion of good knowledge)

q = 1 – p = 0.297

w ( maximum allowable error = 0.05)

n ( sample size) = **706**

**Sample size determination using the power approach**: statcalcal (Epi info 7) was used to calculate the minimum sample size. The sample size calculation was based on the most statistically significant variable in the same topic with similar study, which provides the maximum sample size among others. The variables, which were found statistically significant, were age, sex and educational status. However, educational status was the most frequently statistically significant variable reported in most literatures and was taken for the sample size determination. A power of 80%, non- exposed to exposed ratio (r) =1, confidence level = 95% (with a two tailed alpha). Furthermore, design effect of 2 for multistage sampling and non-response of 10% were considered. Taking p1 = 0.75, p2 = 0.28 AOR = 7.8, r = 1:1, n2 = n1xr

n1 = 42, n 2 = 42, the sample size n final become 185. Where, nf = final sample size, p1 = proportion of people with higher educational level having knowledge about glaucoma, p2 = proportion of people with lower level of education who have poor knowledge about glaucoma, r= the ratio of those people who have higher educational status and aware of glaucoma to people who have low educational status and not aware of glaucoma. Similar procedures were followed for the other two factors and provide a sample size of 156 for age and 110 for sex. When we compare the calculated sample sizes above (706, 689, 491, 110, and 185) the maximum was 706. Hence, the minimum sample size for the study was 706 adults.
