# Supplementary material for: Awareness and knowledge of glaucoma and associated factors among adults: a cross sectional study in Gondar Town, Northwest Ethiopia
Source: BMC Ophthalmol. 2017 Aug 24;17:154. doi: 10.1186/s12886-017-0542-z (PMC5571668; doi:10.1186/s12886-017-0542-z)
Supplement: Supplementary file 2 — Sampling technique (DOCX 14 kb) [file 12886_2017_542_MOESM2_ESM.docx]

**Additional file 2: Sampling technique**

**Gondar town**

21 kebeles (N = **53,725** HH)

**Simple Random Sampling**

**Cherikos**

Nc = 2,421HH

**Kilil Eyesus**

Nk=1,245 HH

**Gebreal**

Ng=3574 HH

**Lideta**

Nl = 4771HH

**Dimaza**

Nd= 3,627HH

***Proportional Allocation to Size***

**nc = 109**

**ni=215**

**nd= 164**

**nk = 56**

**ng = 162**

***Systematic Random Sampling***

Additional file 2: Schematic presentation of the sampling technique for the study on awareness and knowledge of glaucoma and associated factors among adults age 35 and above years in Gondar town, Northwest Ethiopia, April 2016
