# Supplementary material for: Awareness and knowledge of glaucoma and associated factors among adults: a cross sectional study in Gondar Town, Northwest Ethiopia
Source: BMC Ophthalmol. 2017 Aug 24;17:154. doi: 10.1186/s12886-017-0542-z (PMC5571668; doi:10.1186/s12886-017-0542-z)
Supplement: Supplementary file 3 — English version of questionnaire (DOCX 26 kb) [file 12886_2017_542_MOESM3_ESM.docx]

**Additional file 3: English version of questionnaire**

**Part one: Socio – demographic data**

| S.No. | Question | Response | Code |
| --- | --- | --- | --- |
| 101 | Age | _____years |  |
| 102 | Sex | 1. Male 2. Female |  |
| 103 | Religion | 1. Orthodox 2. Muslim 3. Protestant 4. Catholic 5. Other(specify)______ |  |
| 104 | Ethnicity | 1. Amhara 2. Oromo 3. Tigrie 4. Kmant 5. Others (specify)___________ |  |
| 105 | Marital status | 1. Single 2. Married 3. Divorce 4. Widowed |  |
| 106 | Educational status | 1. Unable to read and write 2. Read and write only 3. 1-4 grade 4. 5-8 grade 5. 9-12 grade 6. College and above |  |
| 107 | Type of type of occupation | 1. Farmer 2. Housewife 3. Government employee 4. Merchant 5. Others(specify)___ |  |
| 108 | Average household monthly income (ETB) |  |  |

**Part two: Awareness of glaucoma, general & eye health related data**

| 201 | Have you ever heard of glaucoma? | - 1. Yes   2. No (go to Q 206) | Code |
| --- | --- | --- | --- |
| 202 | Where did you first hear about glaucoma?  (Check all possible responses) | 1. News Media 2. Brochures/ posters 3. School 4. Health workers 5. People with glaucoma 6. Other (specify)_______ |  |
| 203 | How would you explain glaucoma? | 1. High eye pressure damaging eye 2. Causes damage to the eye nerve 3. Causing visual field loss 4. Other (specify)________ 5. No explanation given |  |
| 204 | Do you have family member with glaucoma? | 1. Yes 2. No ( go Q 206) 3. Do not know (go to Q 206) |  |
| 205 | If “Yes” who is affected? | 1. Father 2. Mother 3. Sister 4. Brother 5. Other (specify)_____ |  |
| 206 | Have you examined your eyes? | 1. Yes 2. No ( go to 209) |  |
| 207 | When was your last eye exam?  (in months) | ______________ |  |
| 208 | Do have glaucoma? | 1. Yes 2. No 3. Do not know |  |
| 209 | Do you have hypertension currently? | 1. Yes 2. No 3. Do not know |  |
| 210 | Do you have diabetes mellitus currently? | 1. Yes 2. No 3. Do not know |  |

**Part three: knowledge related questions about glaucoma**

| S. No. | Question | Possible response | Code |
| --- | --- | --- | --- |
| 301 | Can glaucoma occur without symptom? | 1. Yes 2. No 3. Do not know |  |
| 302 | What are the risk factors for glaucoma?  ( please check all possible answers) | 1. High eye pressure 2. Old age 3. Family history of glaucoma 4. Other (specify)_______ 5. Do not know |  |
| 303 | Is early blindness from glaucoma preventable? | - 1. Yes   2. No   3. Do not know |  |
| 304 | Is glaucoma curable? | 1. Yes 2. No 3. Do not know |  |
| 305 | When does glaucoma affect vision? | 1. As soon as it start 2. Slowly 3. Do not know |  |
| 306 | Can glaucoma cause blindness? | 1. Yes 2. No 3. Do not know |  |
| 307 | Is glaucoma treatable? | 1. Yes 2. No ( go to 210) 3. Do not know ( go to 210) |  |
| 308 | What treatments do you know?  (Check all that are mentioned) | 1. Medicines – eye drops 2. Surgery 3. Radiation/laser 4. Other------------------ 5. Do not know |  |
| 309 | What is the purpose glaucoma treatment? | 1. To restore vision 2. To delay progression 3. Other (specify)______ 4. Do not know |  |
| 310 | Is glaucoma damage reversible via treatment? | 1. Yes 2. No 3. Do not know |  |
| 311 | Is glaucoma curable? | 1. Yes 2. No 3. Do not know |  |
| 312 | Is glaucoma heritable from families? | 1. Yes 2. No 3. Do not know |  |
| 313 | Are you at risk of glaucoma? | - 1. Yes   2. No   3. Do not know |  |
| 314 | If “Yes” for Q 213, Why | ____________________________ |  |
