# Supplementary material for: Awareness and knowledge of glaucoma and associated factors among adults: a cross sectional study in Gondar Town, Northwest Ethiopia
Source: BMC Ophthalmol. 2017 Aug 24;17:154. doi: 10.1186/s12886-017-0542-z (PMC5571668; doi:10.1186/s12886-017-0542-z)
Supplement: Supplementary file 4 — Data collection procedures (DOCX 13 kb) [file 12886_2017_542_MOESM4_ESM.docx]

**Additional file 4: Data collection procedures**

Household

Not Eligible

Eligible participants consented to participate in the study

Socio – demographic data and other relevant dta

Go to immediate next household **demographic data**

Have you heard of glaucoma?

No

Yes

Considered as glaucoma unaware and stop asking about knowledge

Knowledge questions

Complete the questionnaire tells some information about glaucoma and thank participant

Additional file 4: Schematic presentation of data collection procedure for the study on awarness and knowledge and associated factors among adults in gondar town, Northwest ethiopia, April 2016
